# Primary Dedifferentiated Duodenal Liposarcoma With Gastric Outlet Obstruction: A Rare Small Bowel Tumor

**DOI:** 10.7759/cureus.50978

**Published:** 2023-12-23

**Authors:** Ali Tariq Alvi, Murali Shankar

**Affiliations:** 1 Internal Medicine, HCA Florida Westside Hospital, Plantation, USA

**Keywords:** melena, small bowel tumor, gastric outlet obstruction, liposarcoma, duodenum

## Abstract

Liposarcoma is the most prevalent malignant soft tissue tumor and is primarily found in extremities and retroperitoneum, but its occurrence within abdominal viscera is rare. Most of these cases have been reported in the esophagus and stomach. Among liposarcomas of the gastrointestinal tract, primary duodenal liposarcomas are exceptionally rare, with only five documented cases in the literature. We describe a case of a 66-year-old female presenting with syncope, melena, and anemia. Computed tomography (CT) showed soft tissue mass involving the superior wall of the duodenum and hypo-enhancing hepatic mass. An endoscopy was performed, which revealed a duodenal mass causing gastric outlet obstruction and significant bleeding. Therefore, laparotomy was performed with extensive lysis of adhesions and gastrojejunostomy.

## Introduction

Adenocarcinomas and neuroendocrine tumors are the most frequently encountered malignancies involving the small bowel, with lymphomas and sarcomas being less common. Among intestinal sarcomas, gastrointestinal stromal tumors (GISTs) are the predominant subtype [1]. Sarcomas comprise only 1% of all malignancies in adults, and among the various types of soft tissue sarcomas, liposarcomas are the most common [2]. According to the World Health Organization (WHO), there are five distinct subtypes of liposarcomas distinguished based on their pathologic features: dedifferentiated liposarcoma, well-differentiated liposarcoma, pleomorphic liposarcoma, round cell liposarcoma, and myxoid liposarcoma [3].

Liposarcomas are commonly found in the extremities and retroperitoneum, whereas primary gastrointestinal liposarcomas are rare [1]. In the gastrointestinal tract, these are primarily found in the esophagus, followed by the stomach, colon, and gastroesophageal junction [4]. Primary duodenal liposarcomas are observed to be the rarest, with only a few cases reported in the literature. Hence, we present a case of primary dedifferentiated liposarcoma in the duodenum with a complication of gastric outlet obstruction and significant gastrointestinal hemorrhage.

## Case presentation

**Figure 1:**
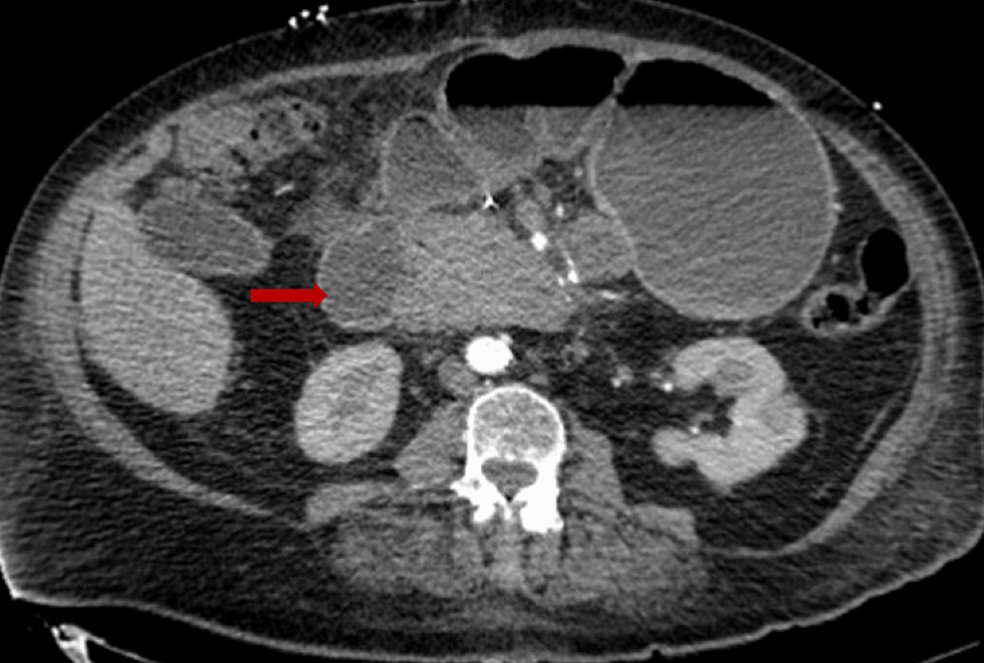
Axial view of computed tomography (CT) of the abdomen demonstrating a lobular soft tissue mass involving the duodenum (red arrow)

We describe a 66-year-old female with a medical history of essential hypertension, hyperlipidemia, and hysterectomy who presented in the emergency department with a syncopal episode and melena. In the ER, she was afebrile, with a heart rate of 99 beats per minute, blood pressure of 104/53 mmHg, and oxygen saturation of 100% on room air. She denied hematemesis, hematochezia, abdominal pain, nausea, and vomiting. Her laboratory investigations were significant for hemoglobin of 5.2 g/dl. Computed tomography (CT) showed a lobular soft tissue mass involving the superior wall of the duodenum measuring approximately 6.5 cm x 4.7 cm, a large adjacent mesenteric lymph node measuring 2.4 cm in diameter, and a subtle hypo-enhancing hepatic mass measuring 4.0 cm x 3.0 cm (Figure 1).

**Figure 2:**
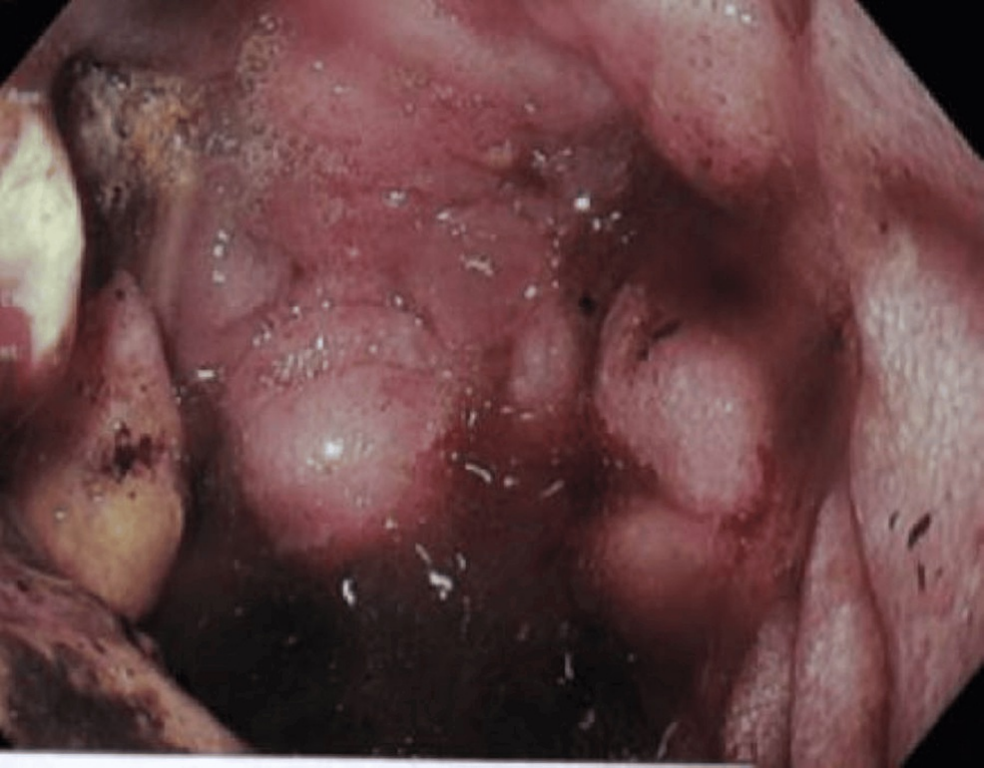
Endoscopy (EGD) showing the second part of the duodenum with a necrotic ulcer, profuse bleeding, and duodenal obstruction

An endoscopy was performed, which revealed a duodenal mass with significant bleeding and gastric outlet obstruction (Figure 2).

**Figure 3:**
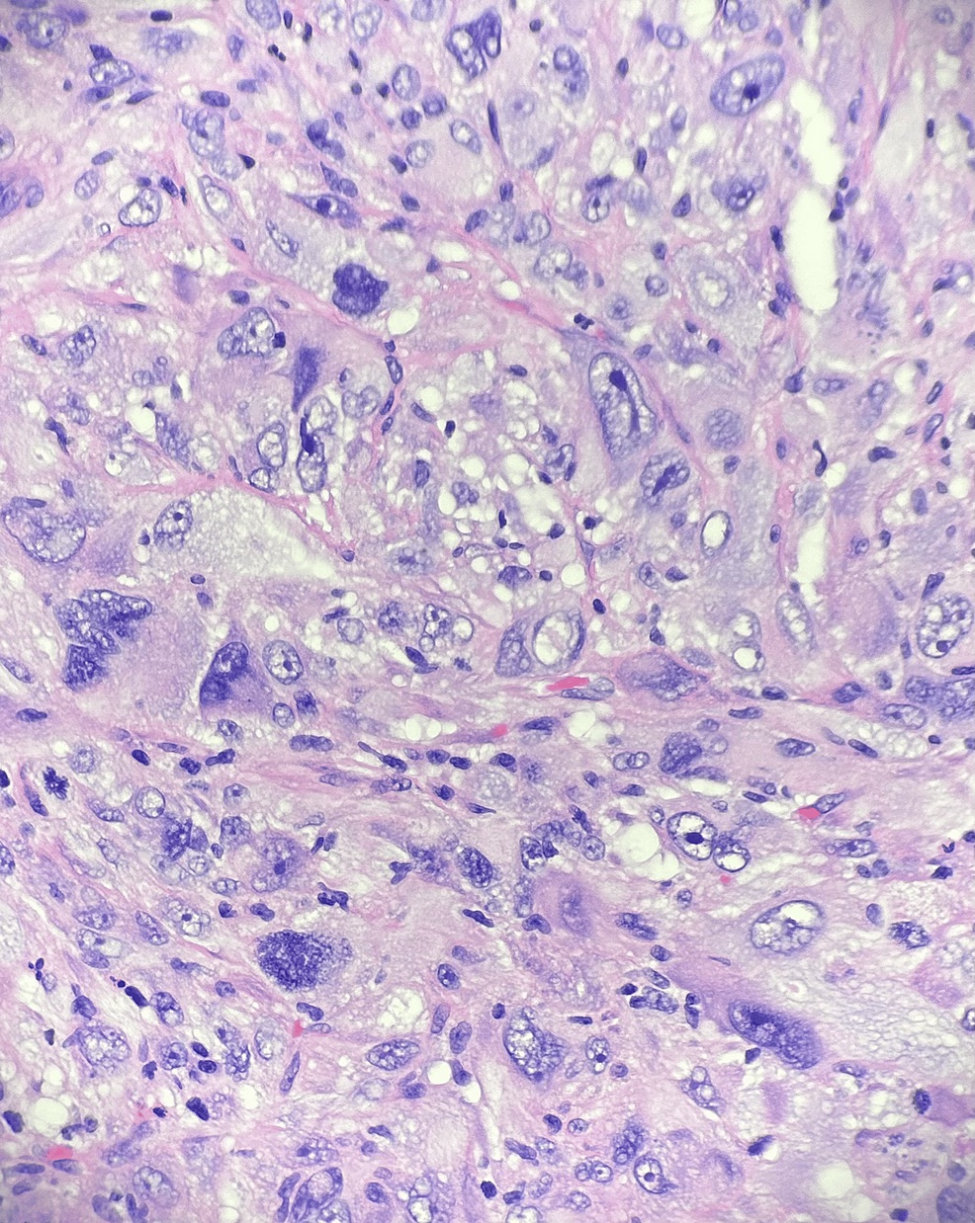
Histopathology of the tissue demonstrating atypical spindle cells and adipocytes

It was followed by the embolization of small mesenteric collateral originating from the proximal splenic artery coursing toward the duodenal mass in an attempt to stop bleeding. Due to gastric outlet obstruction and persistent bleeding, laparotomy was performed with extensive lysis of adhesions and gastrojejunostomy. Histopathology was consistent with high-grade pleomorphic sarcoma consistent with dedifferentiated liposarcoma (Figure 3).

## Discussion

The most common subtypes within liposarcomas are well-differentiated and dedifferentiated liposarcomas, and amplifications in the 12q13-15 chromosomal region characterize both. Nevertheless, the recurrence rate is high with dedifferentiated liposarcomas [5]. The term dedifferentiated liposarcoma was initially coined in 1979 [6]. It can arise from existing well-differentiated liposarcoma in 10% of cases or originate de novo in 90%. Dedifferentiated liposarcoma can be characterized by a well-differentiated liposarcoma and non-lipogenic sarcoma-like component with diverse histologic grades [7].

The presenting symptoms associated with primary duodenal liposarcoma vary depending on tumor size, location within the duodenum, and potential complications like obstruction and compression of surrounding organs. These patients can present with nonspecific symptoms like fatigue, weakness, abdominal pain, recent weight loss, and abdominal distention, which makes it difficult to diagnose the disease early in the condition [1].

Given the rarity of liposarcomas in the gastrointestinal tract, the recommendations for management are derived from those applied to retroperitoneal liposarcomas. The first line of treatment for these soft tissue sarcomas is surgical resection, which is the only treatment modality for a potential cure [4]. The status of surgical margins significantly impacts overall survival, and radiation therapy may improve locoregional control. Nevertheless, this benefit must be carefully weighed against potential side effects of radiotherapy on intra-abdominal organs. Chemotherapy with doxorubicin has been the cornerstone of protocols for managing these sarcomas, with the possibility of considering multiagent regimens on a case-by-case basis [1,8].

There are five documented cases of primary duodenal liposarcoma in English literature, with the first case reported by Okabayashi et al. in 2013. Among the five cases, three were dedifferentiated liposarcomas [1,4,9-11].

## Conclusions

We report a rare case of dedifferentiated liposarcoma arising from the duodenum with gastric outlet obstruction, a highly uncommon location for liposarcomas within the gastrointestinal tract, with only five cases reported. It highlights the importance of carefully distinguishing dedifferentiating liposarcomas from their morphologic counterparts, as the tumor’s extent and aggressiveness may be higher than what is anticipated on clinical assessments. Given the limited number of cases, it is challenging to establish definitive recurrence patterns for gastrointestinal liposarcomas. Nevertheless, pathologists should keep dedifferentiated liposarcomas in their differential diagnoses when encountering high-grade tumors within the duodenum.
